# Terahertz Photons Promote Corneal Injury Repair via Epithelial Proliferation, Migration, and Inflammation Reduction

**DOI:** 10.1167/iovs.67.3.56

**Published:** 2026-03-30

**Authors:** Hao Lin, Hongwei Wu, Chenjie Liu, Yuan Zhong, Jingxue Zhang, Shen Wu, Jiaying Li, Zi-Bing Jin, Chao Chang, Ningli Wang

**Affiliations:** 1 Innovation Laboratory of Terahertz Biophysics, National Innovation Institute of Defense Technology, Beijing, China; 2 Qingdao University, Qingdao, Shandong, China; 3 University of Electronic Science and Technology of China, Chengdu, Sichuan, China; 4 Department of Engineering Physics, Tsinghua University, Beijing, China; 5 Beijing Institute of Ophthalmology, Beijing Tongren Eye Center, Beijing Tongren Hospital, Capital Medical University, Beijing, China; 6 School of Physics, Peking University, Beijing, China; 7 Henan Academy of Innovations in Medical Science, Zhengzhou, Henan, China

**Keywords:** corneal injury repair, alkali burn, corneal epithelial, terahertz photons, inflammation reduction

## Abstract

**Purpose:**

Alkali burn of the cornea is a severe ophthalmic emergency that often results in permanent vision loss due to its destructive impact on corneal tissue. Current treatments face challenges in promoting rapid healing and restoring tissue integrity.

**Methods:**

This study demonstrates a non-contact therapeutic approach using terahertz (THz) photons transmitted via optical fibers for alkali-burned corneas. The effects of THz proton treatment on human corneal epithelial cells (HCECs) were evaluated through proliferation and migration assays. RNA sequencing was used to analyze gene expression changes, and scanning electron microscopy (SEM) was used to observe cellular structural modifications. Murine models of alkali-burned corneas were subjected to continuous local THz photon therapy to assess wound healing, edema, neovascularization, and inflammatory cell infiltration.

**Results:**

THz photon treatment significantly promoted HCEC proliferation and migration. RNA sequencing revealed that THz photons modulated genes involved in energy metabolism, inflammation, and cell growth pathways. SEM images showed enhanced lamellipodia, filopodia, and microvilli formation, explaining the mechanism of cell migration. In vivo, THz proton therapy accelerated corneal epithelial wound healing, reduced edema and neovascularization, decreased inflammatory cell recruitment, and improved tissue integrity.

**Conclusions:**

Terahertz photon therapy represents an effective, non-invasive, and biocompatible approach for treating ocular surface damage from alkali burns, holding promise for clinical translation to improve therapeutic outcomes.

The visual system serves as the primary gateway for human interaction with the external environment, processing approximately 80% of sensory information through complex neuro-optical pathways.^1^ However, the cornea—the outermost protective barrier of the eye—is particularly vulnerable to chemical insults. Recent epidemiological studies indicate that over 300,000 individuals worldwide annually sustain vision-impairing corneal injuries from alkaline or acidic exposures, with incidence rates escalating by 4% to 7% yearly in industrialized regions.^2^ Alarmingly, 40% to 60% of severe chemical burns progress to corneal opacification or neovascularization, frequently culminating in irreversible blindness despite maximal medical intervention.^3^^,^^4^ Current therapeutic paradigms, including amniotic membrane transplantation and keratoprostheses, remain constrained by donor tissue shortages, immunological rejection risks, and limited capacity to restore native corneal transparency.^3^^,^^5^ Emerging insights into photothermal and chemodynamic stromal remodeling therapy have revitalized interest in targeted regenerative strategies.^6^^–^^9^ Nevertheless, the pathophysiological complexity of chemical burns—simultaneously involving epithelial loss, imbalance of immune microenvironment, and dysregulated neurotrophic signaling—continues to challenge conventional monotherapeutic approaches.

Over the past decade, photon-based therapeutic modalities, particularly terahertz (THz) photon therapy, have gained substantial traction in clinical medicine due to their diagnostic and therapeutic potential.^10^^–^^13^ The biological significance of THz irradiation in maintaining cellular homeostasis and stimulating neurogenesis has been extensively documented,^14^^–^^16^ with emerging translational applications employing optical fiber-mediated photon delivery for neural regeneration therapies.^17^^–^^19^ Notably, the non-thermal bioeffects of THz radiation demonstrate marked anti-inflammatory responses across diverse cellular systems, suggesting its potential as a repeatable, minimally invasive intervention for ocular surface rehabilitation.^20^^,^^21^

Building upon these foundations, we present a novel ophthalmic therapeutic paradigm utilizing THz photon irradiation. Through systematic evaluation in an alkali burn–induced acute ocular injury murine model (Fig. 1), this investigation established preliminary safety profiles and quantified the therapeutic efficacy parameters for this innovative approach.

**Figure 1.**
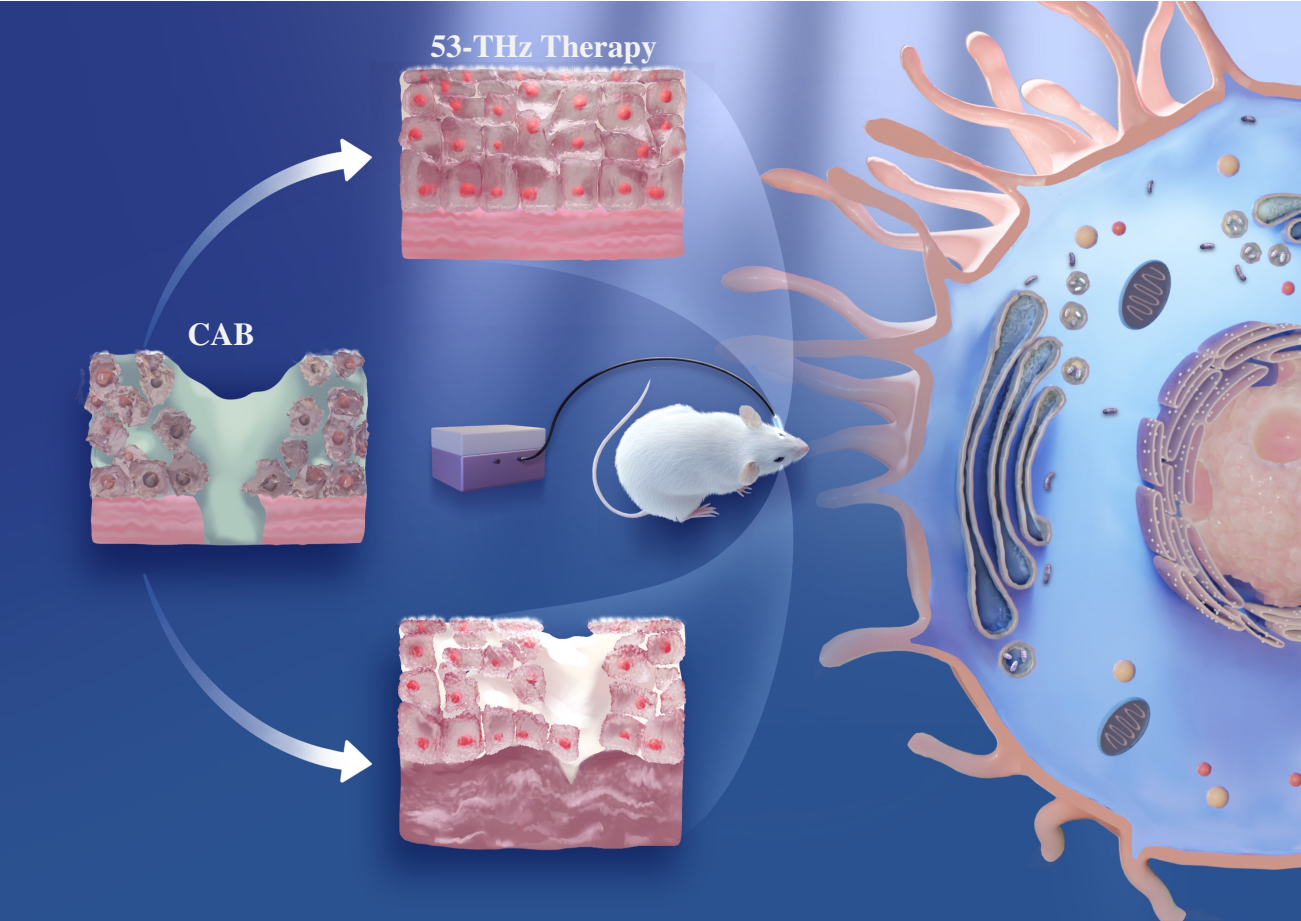
Effects of THz proton therapy on murine corneal alkali burns (CABs). THz proton therapy mitigates the prognosis of corneal alkali burns by promoting the proliferation and migration of HCECs.

## Methods

### THz Proton Sources

THz waves were emitted by a commercial mid-infrared laser source. The operating frequencies of the laser source were set at 42 THz, 49 THz, and 53 THz. We designed switchable optical paths for delivering THz photon treatment to cells seeded in culture dishes via spatial light and to animal ocular surface tissues via optical fibers.^22^

### Cell Culture

Immortalized human corneal epithelial cells (HCECs; CL-0743; Procell, Wuhan, China) were cultured in Gibco Minimum Essential Medium (MEM) (11095080; Thermo Fisher Scientific, Waltham, MA, USA) supplemented with Gibco 10% fetal bovine serum (A5669901; Thermo Fisher Scientific) and 1% penicillin–streptomycin. Cells were maintained in a 37°C incubator with a 5% CO_2_ atmosphere. Unlike culture vessels used in other experiments, we selected the low µ-Dish (80136; ibidi GmbH, Gräfelfing, Germany) for THz proton treatment. During cell passage, 500 µL of cell suspension was added to the ibidi culture dish. Approximately 2 hours later, after confirming that cells had adhered to the dish bottom, 500 µL of complete medium was added.

### THz Photon Treatment of Cells

**Figure 2.**
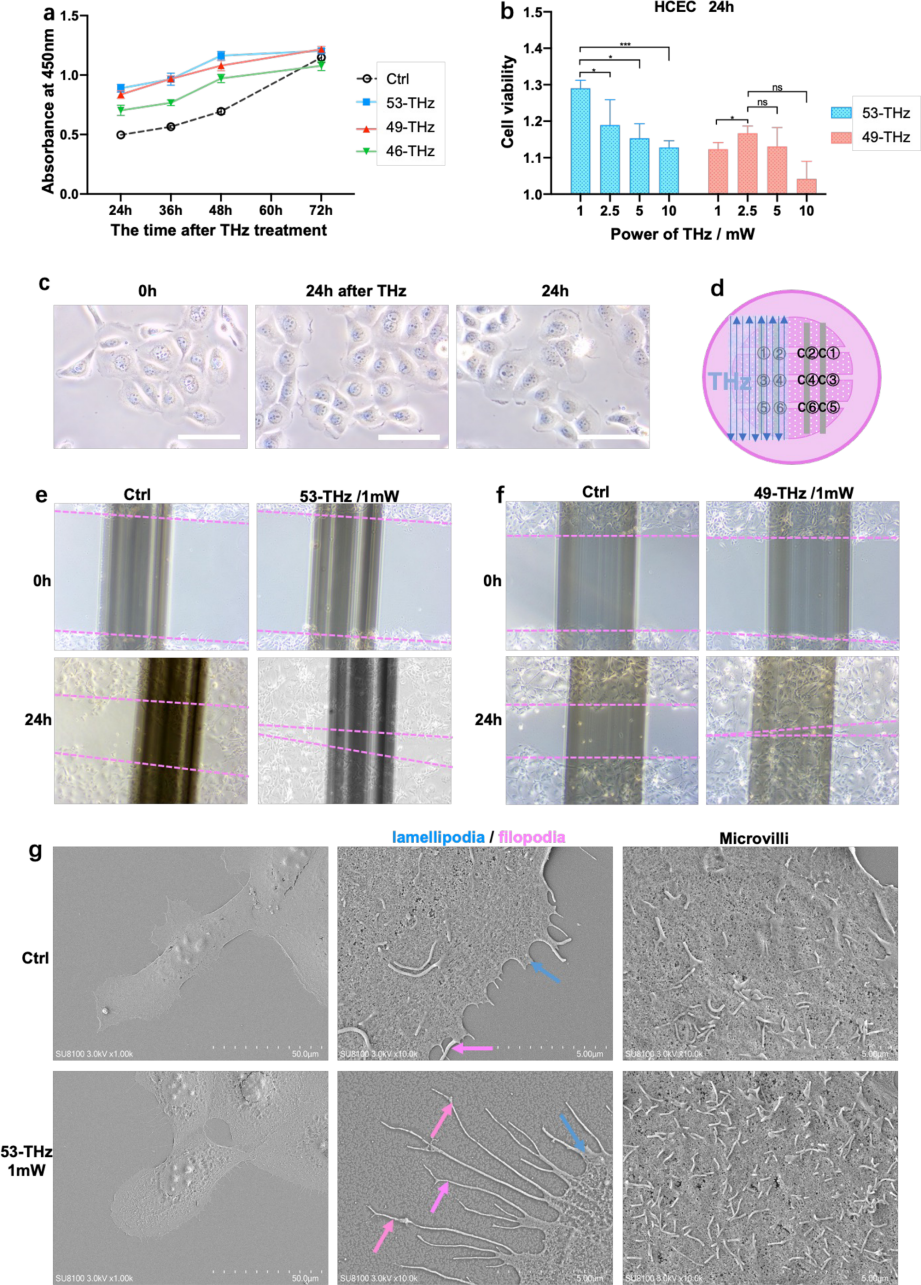
The proliferation and migration of HCECs after THz proton treatment. (**a**) Proliferation experiment of HCECs at 24, 36, 48, 60, and 72 hours after 46-THz, 49-THz, and 53-THz irradiation. (**b**) The viability of HCEC at 24 hours after irradiation exposures of varying power. Cell viability was normalized and is presented with the absorbance at 450 nm of the control group (without THz irradiation) set as 1.0. Data are mean ± SEM (*n* = 6/group). (**c**) The morphology of HCECs seeded in ibidi dishes before and after THz irradiation. *Scale bar*: 50 µm (magnification, 200×). (**d**) HCECs seeded in the same ibidi dish were scanned in sections by controlling the switch of THz source. (**e**) The morphology of HCECs after 53-THz (1-mW) irradiation and sham irradiation (magnification, 40×). (**f**) The morphology of HCECs after 49-THz (1-mW) irradiation and sham irradiation (magnification, 40×). (**g**) The changes of cell surface were observed by SEM. *Scale* *bars* for HCECs were 50 and 5 µm (magnification, 1000× and 10,000×).

Low-walled culture dishes were placed in hollow plastic trays to receive THz photon treatment.^22^ THz photons emitted by the laser were reflected by optical mirrors onto the dish bottom, forming a light spot with a diameter of 400 µm. To test the effects of different THz photons on corneal cell proliferation, we designed a scanning protocol targeting the bottom area of the ibidi culture dish. Through the movement of the plastic tray, cells attached to the dish bottom received treatment from the fixed THz light spot. To verify the effect of THz photon treatment on cell migration, we innovatively designed a specialized scanning protocol, as shown in Figure 2d, where the two sides of the same cell scratch were treated with or without THz photons. Subsequently, cells were incubated in an appropriate environment for 24 hours for observation and imaging.

### CCK-8 Assay

To evaluate cell viability, THz-treated cells were detached from the culture dishes and seeded in 96-well plates at a density of 1000 cells per well. Cell proliferation was detected using the CCK-8 Assay Kit (HY-K0301; MedChemExpress, Monmouth Junction, NJ, USA). At 24, 36, 48, 60, and 72 hours after THz photon treatment, the absorbance of the culture medium at 450 nm was measured to calculate the proliferation of HCECs. Cell viability of HCECs was normalized and presented with the absorbance value at 450 nm of the control group (without THz irradiation) set as 1.0.

### Scanning Electron Microscope

HCECs were fixed by 2.5% glutaraldehyde at 4°C for 2 hours. The samples were mixed with 1% (v/v) osmium tetroxide at 4°C for 1 hour. Subsequently, samples were coated with gold and observed under scanning electron microscopy (SEM; VEGA3; Servicebio, Hubei, China).

### RNA Sequencing

Total RNA was extracted from HCECs using Trizol reagent. Subsequent library construction was performed with the TruSeq Stranded mRNA LT Sample Prep Kit (Illumina, San Diego, CA, USA). Transcriptome sequencing and associated analyses were conducted by OE Biotech Co., Ltd. (Shanghai, China). These libraries were sequenced on an Illumina HiSeq X Ten platform, yielding 150-bp paired-end reads.^23^ Clean reads were aligned to the human genome using HISAT2.^24^ The fragments per kilobase of transcript per million mapped reads (FPKM) values for each gene were computed using Cufflinks, and read counts for each gene were obtained via HTSeq-count.^25^ Differential expression analysis was carried out using the DESeq R package (R Foundation for Statistical Computing, Vienna, Austria), with the thresholds for significant differential expression set at *P* < 0.05 and |log_2_ fold change| > 1. Hierarchical clustering analysis of differentially expressed genes (DEGs) was performed to visualize the gene expression patterns across different groups and samples. Gene Ontology (GO) enrichment and Kyoto Encyclopedia of Genes and Genomes (KEGG) pathway enrichment analyses of DEGs were implemented in R based on the hypergeometric distribution.^26^

### Animals

Eight-week-old female mice were used in this study to evaluate the safety of THz photon therapy and establish an animal model of ocular surface injury. Six-week-old female mice were acclimated for 2 weeks in an environment with appropriate temperature, humidity, water, and food before treatment. The experimental procedures and protocols in this study were approved by the Animal Ethics Review Committee of Beijing Tongren Hospital, Capital Medical University (TRLAWEC2023-03), and all animal usage complied with the ARVO Statement for the Use of Animals in Ophthalmic and Vision Research.

### Corneal Alkali Burn Model

Mice were anesthetized with 3.5% isoflurane gas. A 3-mm-diameter filter paper soaked with 1-mol/L NaOH was applied to the central area of the cornea and removed after 1 minute. The cornea was immediately rinsed thoroughly with 30 mL of 0.9% normal saline. After alkali burn, 5 µL of antibacterial eye drops (levofloxacin 5 mg/mL; Santen Pharmaceutical, Osaka, Japan) was administered twice daily to prevent accidental infection until the mice were euthanized. All corneal alkali burn injuries were induced in the right eye. Mice were randomly divided into the control group (CAB) and the THz photon therapy group (CAB+THz).

### THz Photon Therapy of Animals

For the in vivo experiments, a THz laser with a frequency of 53 THz was used. THz photons were focused by a THz lens and coupled into an optical fiber.^15^ To deliver THz photons to specific corneal regions, THz therapy was performed under microscopic guidance (Figs. 4a, 4b). The injured corneal area of mice received precise THz therapy for 5 minutes each time, twice daily. To observe the expression of cytokines after corneal injury, mice were euthanized on day 11, and corneal samples were collected. To evaluate corneal fibrosis caused by alkali burn, researchers assessed corneal opacity and haze daily using slit-lamp photography and a scoring system. The grading system was as follows: 0, no opacity with a completely transparent cornea; 1, mild opacity with the iris and lens visible; 2, moderate opacity with the iris and pupil still detectable; 3, severe opacity with the pupil barely visible; and 4, complete opacity with the pupil invisible. Mice were euthanized on day 21, and corneal samples were examined.

### Immunofluorescence and Imaging

The completely enucleated mouse eyeballs were embedded in Tissue-Tek O.C.T. Compound (4583; Sakura Finetek, Torrance, CA, USA), followed by quick freezing in liquid nitrogen to fix their morphology. The eyeballs were sectioned into 12-µm slices using a cryostat (CM1520; Leica, Wetzlar, Germany) and rapidly attached to anti-slip glass slides, then air-dried at room temperature for 2 hours. The sample slides were immersed in acetone for 5 minutes for fixation, followed by washing twice with PBS. The corneal samples were blocked for 1 hour at room temperature with a buffer containing goat serum (Solarbio, Beijing, China) and 0.1% Triton X-100 (9036195; Sigma-Aldrich, St. Louis, MO, USA). They were incubated overnight at 4°C in primary antibody solutions, then washed three times with PBS. Subsequently, they were incubated with fluorescein isothiocyanate (FITC)- or Cy3-conjugated secondary antibodies (Elabscience, Wuhan, China) at room temperature for 2 hours. After three washes with PBS, the sections were finally mounted using an antifade mounting medium (HY-K1042; MedChemExpress).^26^ Primary antibodies used for immunofluorescence (IF) included anti-α-SMA (AF1032, 1:400 dilution; Affinity Biosciences, Jiangsu, China) and anti-IL-1β (AF5103, 1:400 dilution; Affinity Biosciences). All fluorescence images were captured using a Leica DMi8 fluorescent microscope. The central corneal thickness of mouse corneal sections was quantified using the built-in software of the Leica microscope imaging system. The data were expressed as mean ± SD, and the significance of differences was analyzed by *t*-test.

### Real-Time Reverse-Transcription Polymerase Chain Reaction

The CAB group and CAB+THz group corneas were removed at 11 days. Each murine cornea was treated as an individual sample and preserved in 0.05 mL of physiological saline. The corneal tissues were then homogenized using an ultrasonic disruptor while being kept on ice. RNA was extracted from each corneal tissue sample using an RNA extraction kit (RC102; Vazyme, Nanjing, China), and its concentration was measured using a NanoDrop One (Thermo Fisher Scientific). Following the instructions of the Vazyme reverse transcription kit (R323), the corneal RNA was reverse transcribed. Murine primers were designed and synthesized (AgBio, Shanghai, China), after which PCR analysis was performed. The reagents used included diethyl pyrocarbonate (DEPC) water (G3004; Servicebio) and Taq Pro Universal SYBR qPCR Master Mix (Q712; Vazyme). *β-Actin* was used as an internal control gene.^27^

## Results

### THz Proton Therapy Promotes the Proliferation of HCECs

To investigate the proliferation-promoting effect of THz photons on HCECs, immortalized HCECs were co-cultured in ibidi dishes to receive THz photon treatment. Morphologies of CECs were captured, and the CCK-8 test was conducted afterward (Fig. 2). After 49-THz and 53-THz irradiation, the number of cells increased significantly, and there was slightly weaker increase in the proliferation effect on HCECs after 46-THz irradiation (Fig. 2a; Supplementary Fig. S1). THz power has a direct effect on cell proliferation. The results showed that 1- and 2.5-mW 53-THz irradiation significantly promoted the proliferation of corneal epithelium cells (Fig. 2b). The HCECs after sham irradiation showed better proliferation at 24 hours, with greater cell numbers and a tendency to be congregated. At the same time point after 53-THz treatment (1-mW), the cells had overgrown the bottom of the culture plate, and the cells were tightly connected (Fig. 2c). The CCK-8 test also showed that 53 THz can increase cell viability at 24 hours (Fig. 2b).

### THz Proton Therapy Promotes the Migration of HCECs and Mediates Differential Gene Expression in CECs

The cleft region where the cells grew was established and starved, and THz photons were irradiated to the specific region (Fig. 2d). The changes of cleft width in the irradiated and sham irradiated regions were measured. The results showed that the migration speed of HCECs was increased after one pass of 1-mW 53-THz and 49-THz irradiation (Figs. 2e, 2f). After 24 hours of 53-THz/1-mW photon intervention, the cell morphology and surface structure were observed by SEM. The results showed that THz proton therapy promoted the numbers of cell divisions and the formation of pseudopodia on the cell surface (Fig. 2g), explaining the results of cell migration promoted by THz proton therapy. At the same time, the density of CEC microvilli in the THz treatment group increased, and the morphology was stable (Fig. 2g), suggesting that the THz treatment had a positive regulatory effect on the migration function of corneal epithelium.

**Figure 3.**
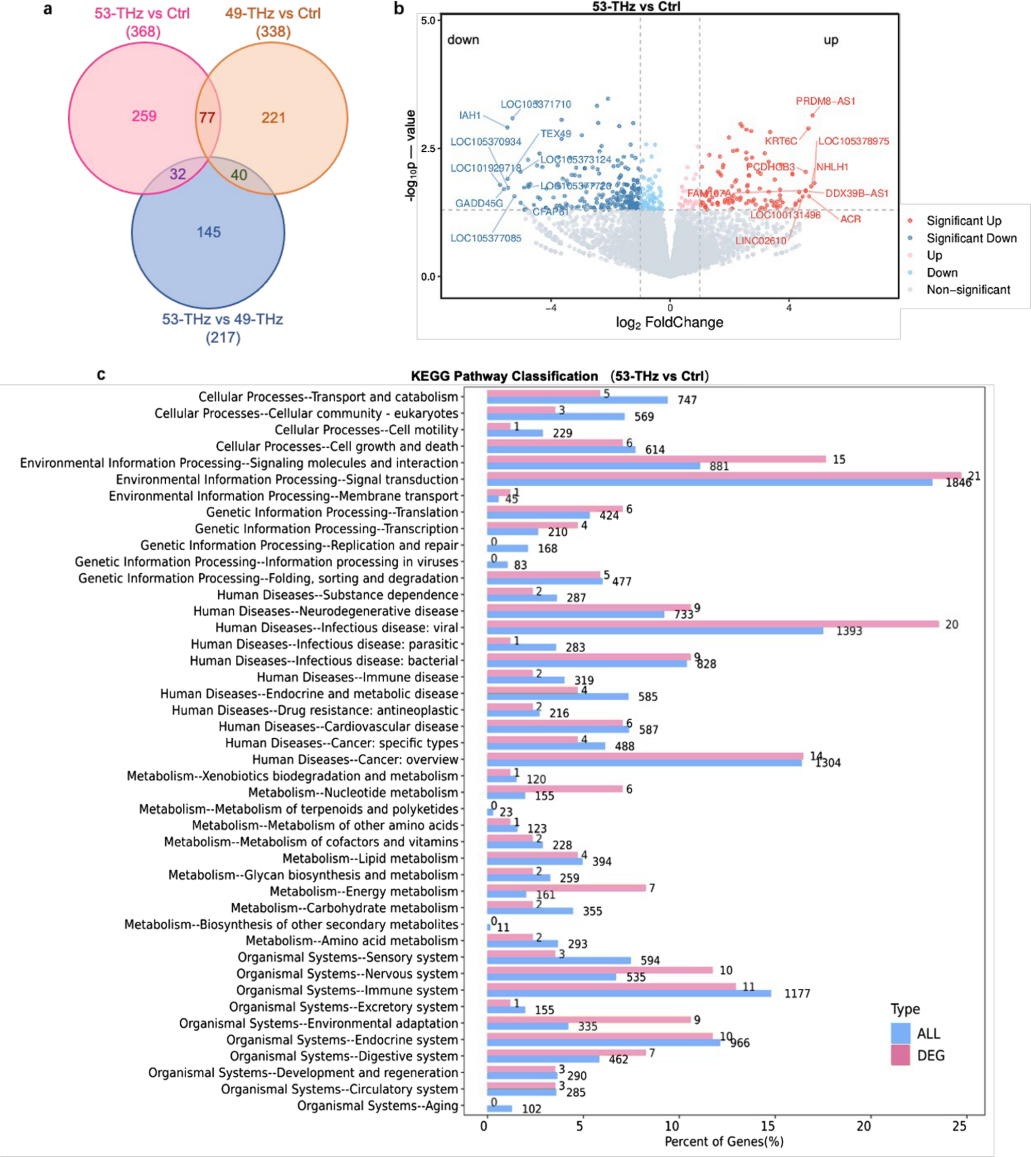
mRNA sequence of HCECs. (**a**) Venn diagram showing DEGs in HCEC comparing the 1-mW 53-THz group versus the control group (53 THz vs. Ctrl), the 1-mW 49-THz group versus the control group (49 THz vs. Ctrl), and the 1-mW 53-THz group versus the 1-mW 49-THz group (53 THz vs. 49 THz). (**b**) Volcano plots for DEGs. (**c**) KEGG pathway classification of DEGs between the control group and the 53-THz group.

mRNA sequencing was used to investigate the biological mechanisms of THz photons, and 368 and 338 DEGs were identified in the 53-THz and 49-THz groups, respectively, with 77 DEGs commonly regulated by the two (Figs. 3a, 3b). KEGG enrichment analysis of 53-THz–regulated DEGs showed that these genes were mainly enriched in cell growth, energy metabolism, nerve growth, and immune regulation pathways (Fig. 3c), which was consistent with our in vitro observation that 53-THz treatment promoted HCEC proliferation and migration.

### Safety and Efficacy of THz Photon Therapy in Mitigating Corneal Alkali Burn

**Figure 4.**
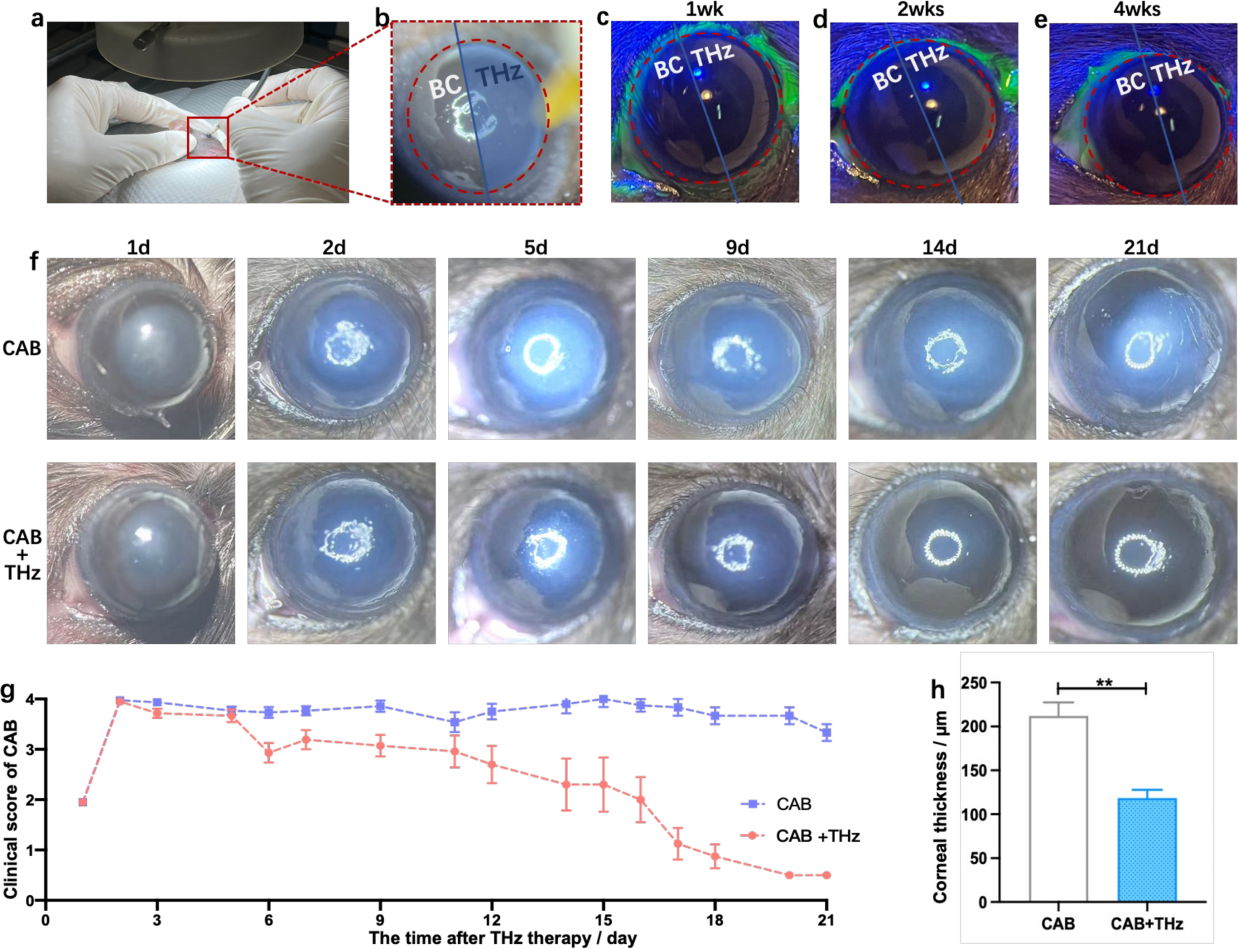
Safety and efficacy of THz photon therapy in mitigating corneal alkali burn. (**a**) THz proton therapy was performed on murine corneas under a microscope. (**b**) The use of a light shield ensured that the contralateral cornea was not exposed to THz irradiation. The cornea was divided into a BC area and THz irradiation area (THz). (**c**–**e**) After 1 week, 2 weeks, and 4 weeks of THz proton treatment, damaged corneas were recovered. THz proton therapy induced a visible wound healing response in the cornea after alkali burn. (**f**, **g**) Representative slit-lamp images (**f**) and opacity score (**g**) of the sham irradiation group (CAB) and THz proton therapy group at 21days. Data are mean ± SEM (*n* = 8/group). (**h**) Statistical analysis of central corneal thickness at 11 days post-treatment (*n* = 6; *P* < 0.01).

To validate the safety of terahertz photon therapy, we treated distinct regions of the cornea in a single mouse with the therapy and left corresponding regions untreated. Precise THz irradiation was delivered to different areas of the murine cornea under a microscope (Figs. 4a, 4b). With the protection of a light shield, the cornea was divided centrally into the blank control (BC) area and the THz-irradiated area (Fig. 4b). The THz-irradiated area was given 10-minute THz irradiation twice daily through an optical fiber. The corneal fluorescein sodium staining test was used to evaluate corneal damage. Yellow fluorescence could be observed under cobalt blue light in the defect area of the corneal epithelium (Supplementary Fig. S2a), whereas no fluorescence was observed in the intact corneal epithelium (Supplementary Fig. S2b). After 4 weeks of THz photon exposure, there was no significant difference between the THz area and the BC area on the murine ocular surface (Figs. 4c–4e).

To further investigate the therapeutic efficacy of THz photon therapy, a THz light spot of appropriate size (2 mm) was applied to the right eyes of mice following alkali burn induction. The therapeutic outcomes were monitored consecutively for 21 days using a clinical slit-lamp (Fig. 4f). In comparison to the control group, the THz-treated group exhibited significant therapeutic effects after alkali-induced corneal injury. From day 6 to day 21, the clinical scores in the THz group were consistently lower than those in the control group (Fig. 4g). Notably, on day 5, corneas receiving THz photon treatment demonstrated higher transparency relative to the untreated group (Fig. 4f).

### THz Photon Therapy Reduces Corneal Neovascularization and Inflammation

**Figure 5.**
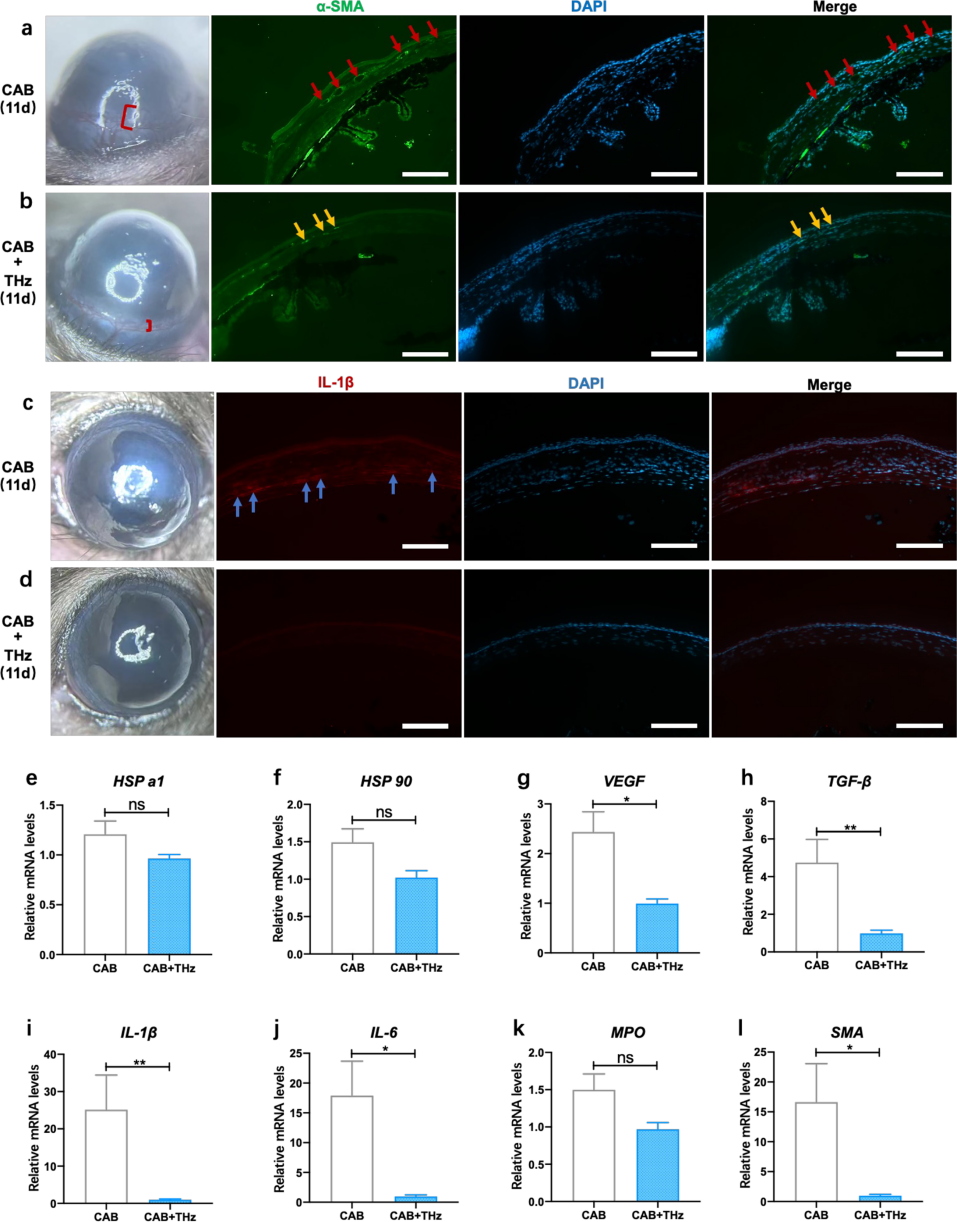
Therapeutic effects of THz on alkali-burned corneas. (**a**–**d**) Representative images of the corneal limbus showing neovascularization marked by SMA (**a**) and inflammation marked by IL-1β (**c**) of the CAB group which were reduced after 11 days of THz proton therapy (**b**, **d**). The *red brackets* denote the regions of neovascularization aggregation in the corneal limbus. *Scale bar*: 200 µm. (**e**–**l**) Corneal mRNA levels of *VEGF* (**g**), *TGF-β* (**h**), *IL-1β* (**i**), *IL-6* (**j**), and *SMA* (**l**) were reduced significantly after 11 days of THz proton therapy, whereas no significant differences were observed in the mRNA levels of *HSPa1* (**e**), *HSP90* (**f**), or *MPO* (**k**) (*P* > 0.05). Data are mean ± SEM analyzed using one-way ANOVA (*n* = 6/group).

When the central corneal region is exposed to external stimuli, such as injury, infection, or hypoxia, an inflammatory response is triggered, recruiting immune cells from the limbal vessels to the injured corneal area. Simultaneously, neovascularization from the limbal vessels spreads toward the central cornea, stimulated by inflammatory cytokines. In this experiment, α-SMA was used to label vascular smooth muscle, and IF images confirmed that the extent of limbal neovascularization and its infiltration depth into the central cornea were reduced in the THz group compared to the CAB group (Figs. 5a, 5b). The neovascularization number and area greatly influence corneal opacity after injury. Additionally, the level of corneal inflammatory factors plays a crucial role in determining corneal transparency and the degree of inflammation caused by alkali burns. Corneal inflammation was elevated at 11 days after the alkali burn (Fig. 5c). However, following THz proton therapy, corneal inflammation was significantly reduced, with the corneal thickness and cell distribution approaching normal levels, and no obvious inflammatory cells were observed (Figs. 4h, 5d). We assessed the RNA expression of several proteins in corneal tissues from two groups of mice using reverse-transcription PCR. The expression levels of heat shock proteins (HSPs) in both sample groups showed no statistically significant differences (Figs. 5e, 5f), indicating that THz treatment does not induce thermal effects on corneal tissues. Furthermore, THz treatment significantly downregulated the expression levels of the inflammatory factor interleukin (Figs. 5i, 5j), which is mediated by alkali burn stimulation, whereas the levels of infection-related myeloperoxidase (MPO) remained unchanged (Fig. 5k). Additionally, in the treated group, the expression levels of vascular endothelial growth factor (*VEGF*), smooth muscle actin (*SMA*), and transforming growth factor-beta (*TGF-β*), which are negatively correlated with corneal transparency, were significantly reduced (Figs. 5g, 5h, 5l).

## Discussion

Accumulating clinical observations and experimental evidence underscore the critical temporal window for early therapeutic intervention following corneal alkali burns.^28^ Timely modulation of corneal epithelial regeneration and inflammatory cascades has been shown to attenuate pathological sequelae including stromal neovascularization and fibrotic scarring, thereby improving long-term corneal transparency outcomes. Building upon mechanistic insights from prior studies,^29^^–^^31^ we postulated that targeted enhancement of CEC motility and proliferation constitutes a pivotal strategy for chemical injury rehabilitation. Our laboratory's foundational work has established the neuroregenerative capacity of THz photon therapy in central nervous system repair models,^14^^,^^15^^,^^32^^–^^34^ prompting its translational application in ocular surface disorders.

To systematically evaluate therapeutic potential, three THz frequencies were screened using in vitro CEC models. This frequency-selection rationale aligns with established biophysical principles: The 53-THz band corresponds to carbonyl group vibrational modes,^35^ a molecular signature previously associated with enhanced fibroblast migration. Intriguingly, analogous pro-migratory effects were replicated in HCECs after THz proton irradiation, with quantitative ultrastructural analysis via SEM revealing THz-induced augmentation of lamellipodial/filopodial protrusions.^36^^,^^37^ Complementary transcriptomic profiling demonstrated THz proton–mediated gene expression modulation, mirroring documented non-thermal genomic regulation patterns.^38^^,^^39^ Notably, THz proton–induced membrane ion channel remodeling may constitute a plausible mechanism driving these phenotypic alterations.^16^^,^^32^

The therapeutic safety profile of optimized THz radiation parameters (energy density, 400 mW/cm^2^; exposure duration, 6 minutes) in animal models supports its viability as a non-invasive regenerative modality.^40^^–^^42^ Through integrated high-throughput sequencing and in vivo validation, we delineated THz proton–mediated anti-inflammatory mechanisms in corneal wound healing—a critical finding given the established role of inflammatory dysregulation in delayed mucosal repair.^43^^–^^46^ Specifically, THz proton therapy attenuated pathologic neovascular extension into the corneal stroma^47^^–^^50^ while downregulating TGF-β–mediated myofibroblast differentiation and aberrant extracellular matrix deposition.^51^ Comparative analysis with bevacizumab–zinc ion therapy^50^ revealed similar efficacy in reducing neovascular density and leukoplakia incidence, as quantified by SMA staining. Although this multimodal investigation advances our understanding of THz photonic therapeutics, clinical translation requires further validation in higher mammalian ocular models and human trials. Concurrent engineering efforts to optimize THz emitter portability and miniaturization remain imperative for practical clinical deployment.

In addition to the long-term therapeutic effect of THz, we observed an immediate and significant improvement in corneal transparency following THz irradiation (Supplementary Fig. S3). We hypothesize that THz waves, beyond promoting CECs, may also enhance the functional activity of CECs, which are closely correlated with corneal edema. This hypothesis represents a direction for our future research.

## Supplementary Material

Supplement 1
