# Supplementary material for: Terahertz Photons Promote Corneal Injury Repair via Epithelial Proliferation, Migration, and Inflammation Reduction
Source: Invest Ophthalmol Vis Sci. 2026 Mar 30;67(3):56. doi: 10.1167/iovs.67.3.56 (PMC13037739; doi:10.1167/iovs.67.3.56)
Supplement: Supplement 1 [file iovs-67-3-56_s001.docx]

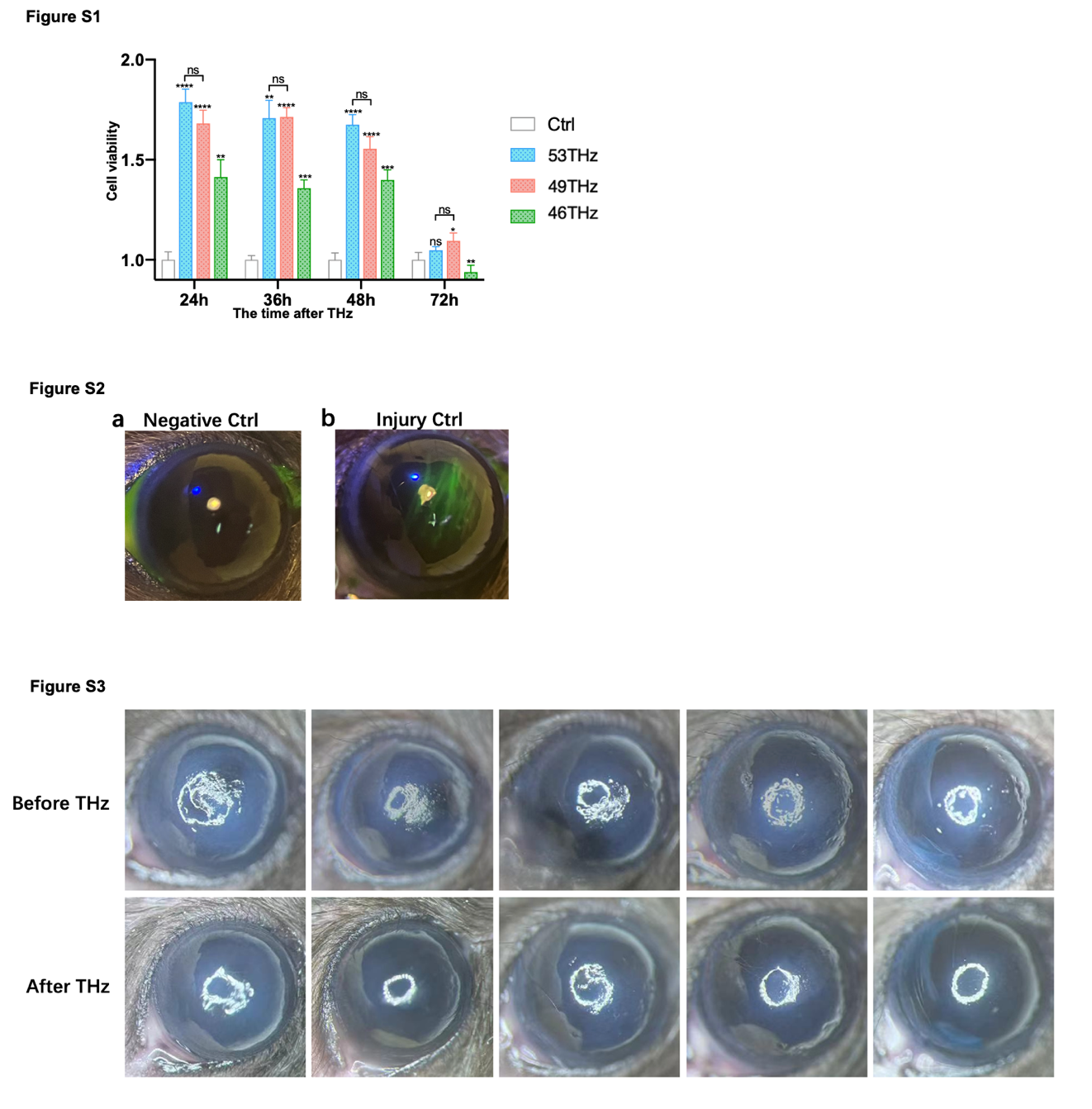


Figure S1. The impact of THz on cell proliferation was the most significant at 24 hours.


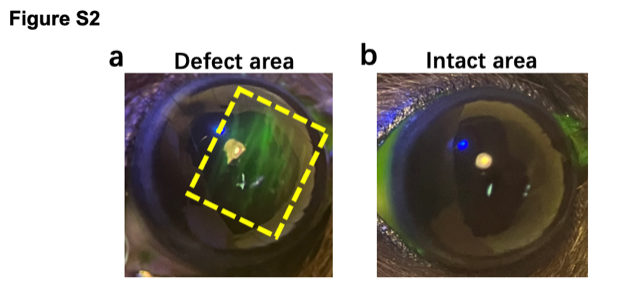


Figure S2. Corneal fluorescein sodium dyeing showed the defect area (a) and intact area (b) of corneal epithelium. The area within the dashed lines indicates the corneal epithelial defect, where yellow fluorescence is visible under cobalt blue light excitation; no fluorescent signal is detected in the intact corneal area.


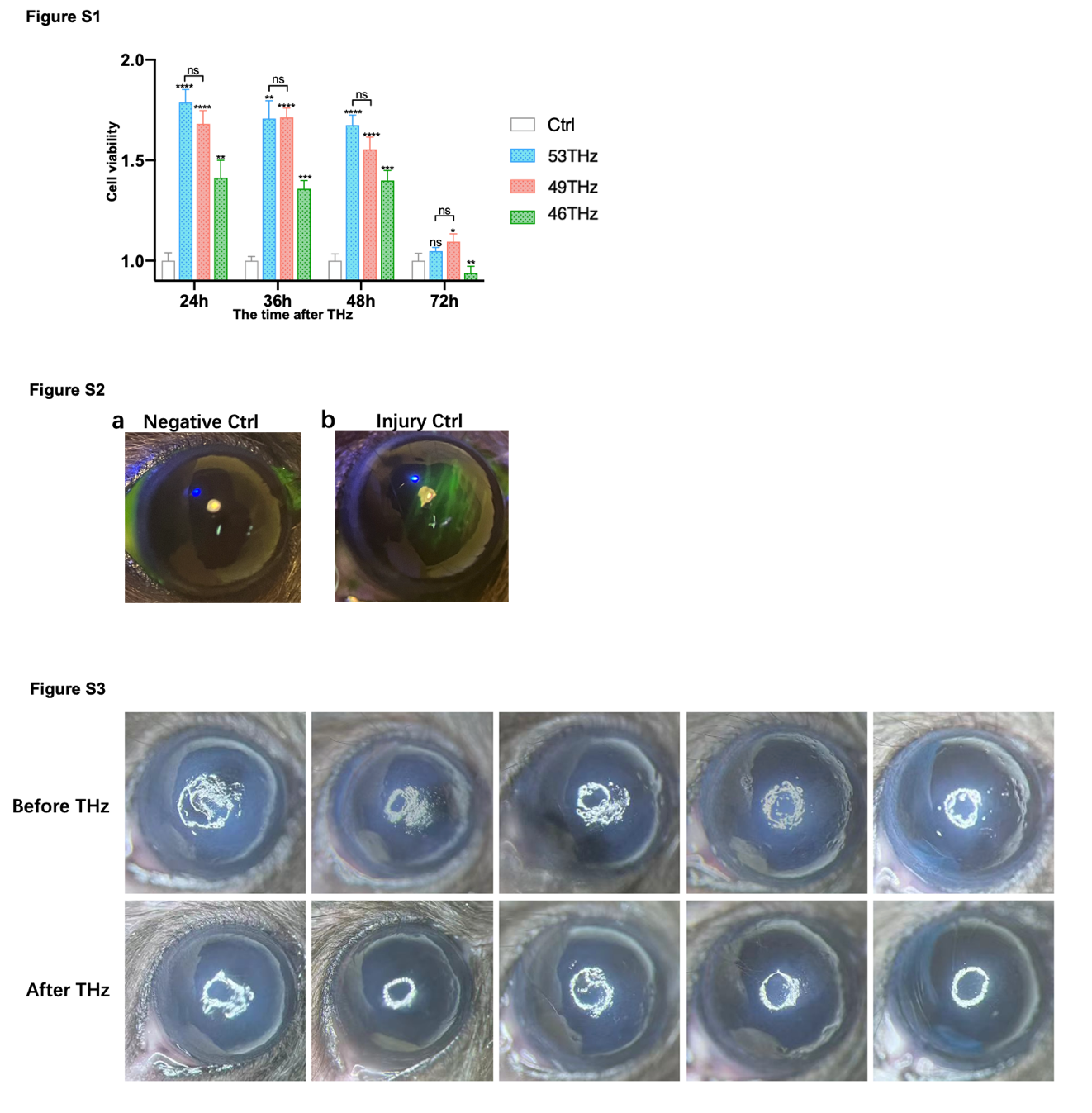


Figure S3. The transparent corneas of mice before and immediately after terahertz photon treatment.
